# Sr and Zr Co-Doped CaCu_3_Ti_4_O_12_ Ceramics with Improved Dielectric Properties

**DOI:** 10.3390/ma15124243

**Published:** 2022-06-15

**Authors:** Yunfei Yu, Qun Wang, Yongqing Li, Mehtab Ur Rehman, Waheed Qamar Khan

**Affiliations:** 1 Faculty of Materials and Manufacturing, Beijing University of Technology, Beijing 100124, China; yuyunfei@emails.bjut.edu.cn (Y.Y.); liyongqing@bjut.edu.cn (Y.L.); mehtaburrehman7@gmail.com (M.U.R.); 2 Institute of Advanced Materials, Bahauddin Zakariya University, Multan 60800, Pakistan; waheedkhan@bzu.edu.pk

**Keywords:** CaCu_3_Ti_4_O_12_ (CCTO), dielectric properties, co-doped, IBLC, mixed valence structure

## Abstract

The dielectric constant of CCTO materials can be as high as 10^4^, which makes it suitable for use in electronic devices but the high dielectric loss limits its application. In this paper, a series of Sr and Zr co-doped CCTO ceramics having the formula Ca_0.8_Sr_0.2_Cu_3_Ti_4−*x*_Zr*_x_*O_12_ (*x* = 0.1, 0.2, 0.3, 0.4) were obtained via a solid-state reaction technique. We force the effect of the Zr content on the phase composition, microstructure, cationic valence states, impedance, and dielectric properties of the as-prepared ceramics to reduce dielectric loss. The results demonstrate that Sr and Zr co-doping increases dielectric constant and reduces dielectric loss simultaneously, and the maximum dielectric constant (1.87 × 10^5^, 1 Hz) and minimum dielectric loss (0.43, 10^2^ Hz) are obtained when *x* = 0.3. Mixed Cu^+^/Cu^2+^ and Ti^3+^/Ti^4+^ valence states are observed to coexist in the co-doped material lattices, which promote dipole polarization, and thereby increase the dielectric constant of the ceramics. The dielectric properties of the materials are analyzed according to the internal barrier layer capacitance model, which elucidates the contributions of the grains and grain boundaries to dielectric performance. The maximum grain boundary resistance (3.7 × 10^5^ Ω) is obtained for *x* = 0.3, which contributes toward the minimum dielectric loss (0.43) obtained for this ceramic at a frequency less than 1 kHz. The average grain sizes of the samples decrease with increasing Zr content, which is the primary factor increasing the grain boundary resistance of the co-doped ceramics.

## 1. Introduction

The rapid development of electronic devices and the continuously decreasing size of devices have generated increasingly intense interest in developing materials with the high dielectric performance required by these devices [1,2,3,4,5]. Among these materials, the cubic ABO_3_ perovskite-type oxide, calcium copper titanate CaCu_3_Ti_4_O_12_ (CCTO), is particularly promising owing to its extremely high dielectric constant that can be as great as 10^4^ and which remains highly stable over a wide temperature range of 100–600 K [6,7]. Accordingly, CCTO materials have become an object of considerable interest [8,9,10] and have currently been applied in a wide variety of electronic devices, such as memory-based devices, capacitors, and electronic switches [11,12].

Nonetheless, the mechanism by which CCTO ceramics attain the colossal dielectric constant remains uncertain. A number of theories have been proposed to address this issue. For example, this has been attributed to a surface barrier layer capacitance (SBLC) effect due to the depletion layer capacitance, similar to the Maxwell–Wagner polarization, between the sample and the electrode [13]. In contrast, the colossal dielectric constant of CCTO ceramics has also been attributed to an internal barrier layer capacitance (IBLC) associated with the insulating grain boundaries positioned between the semiconducting grain structure [14]. Currently, the IBLC model is regarded as the most likely theory for explaining the dielectric properties of CCTO materials [15,16].

However, CCTO materials suffer from high dielectric loss, which currently limits the application value of these materials significantly. Various methods have been applied to reduce the dielectric loss of CCTO materials while retaining their high dielectric constant, such as by adding dopant elements [17,18,19] and modifying the preparation conditions [20,21] or sintering conditions [22,23]. Ouyang et al. [24] researched the sol–gel synthesis of CCTO powders and microwave-sintered CCTO ceramics. A low dielectric loss of 0.16 at 10^3^ Hz was obtained. However, microwave sintering may lead to abnormal crystal growth. Among these, doping strategies represent a particularly effective way of improving the dielectric properties of materials and have been widely applied for CCTO ceramics at the Ca, Cu, and Ti sites of CaCu_3_Ti_4_O_12_. For example, Gaâbel et al. [25] obtained a remarkable decrease in the dielectric loss of CCTO ceramics to a value as low as 0.07 via Ni doping at the Cu site (i.e., CaCu_2.8_Ni_0.2_Ti_4_O_12_). However, the dielectric constant was also significantly reduced. Jesurani et al. [26] investigated the impact of Zr doping at the Ti site on the dielectric properties of CaCu_3_Ti_4−*x*_Zr*_x_*O_12_ (*x* = 0.00, 0.02, 0.10, 0.20, 0.50). The CCTO ceramic material obtained at *x* = 0.1 exhibited a high dielectric constant of about 6020 and a dielectric loss of only 0.52, which was attributed to the particularly dense microstructure of the obtained material. Similarly, Zr^4+^-doped CaCu_3_Ti_4-*x*_Zr*_x_*O_12_ (*x* = 0, 0.05, 0.10, 0.20) ceramics were researched by SU et al. [27]; the dielectric loss was reduced by 55% compared with pure CCTO ceramic at a frequency of 1 kHz. Huang et al. [28] investigated the impact of Sr doping at the Ca site on the dielectric properties of Ca_1−*x*_Sr*_x_*Cu_3_Ti_4_O_12_ (*x* = 0, 0.05, 0.1, 0.2). The dielectric constant was improved, and the maximum value was nearly 3 × 10^4^ at *x* = 0.2.

In addition to single-element doping, co-doping strategies at two of the three sites of CCTO have also been employed to enhance the dielectric properties of CCTO materials. For example, Ren et al. [29] investigated Bi and Al co-doping and showed that when the Bi and Al dopant concentrations were 1 mol%, respectively, the dielectric loss was lower than pure CCTO. Xu et al. [30] applied Y and Zr co-doping at the Ti sites of CCTO, which demonstrated that the co-doping strategy inhibits grain growth to a much greater extent than that observed when applying only a single Y or Zr dopant, and a high dielectric constant (1.0 × 10^4^) with greater temperature stability was thereby obtained while simultaneously decreasing the dielectric loss of the material relative to that of pure CCTO. Espinoza-González et al. [31] applied Sr and La co-doping at the Ca and Cu sites of CCTO, respectively, and the smallest grain size (0.6 μm), the smallest dielectric loss (0.039), and a higher dielectric constant (>10^4^) were obtained by co-doping relative to those obtained when applying only a single Sr or La dopant and for pure CCTO at a high frequency. However, the dielectric properties at a low frequency (<10^3^ Hz) needs to be improved.

While the above discussed results demonstrate that co-doping strategies can achieve greater comprehensive effects on the dielectric properties of CCTO materials, the preparation of CCTO materials with Sr and Zr as co-dopants while changing the Zr doping content has not yet been reported and their effects on dielectric properties are not yet understood. The present work addresses this issue by preparing pure CCTO and a series of Sr and Zr co-doped CCTO ceramics with the formula Ca_0.8_Sr_0.2_Cu_3_Ti_4−*x*_Zr*_x_*O_12_ (*x* = 0.1, 0.2, 0.3, 0.4) via a solid-state reaction technique, and analyzing the effect of the Zr content on the primary factors affecting the dielectric performances of the as-prepared ceramics. The results verify that the Sr and Zr co-doping process increases dielectric constant and reduces dielectric loss simultaneously, and the maximum dielectric constant (1.87 × 10^5^, 1 Hz) and minimum dielectric loss (0.43, 10^2^ Hz) are obtained when *x* = 0.3. Moreover, the mechanism by which colossal dielectric constant and low dielectric loss are obtained is rigorously analyzed according to multiple characterization results and insights derived from the IBLC model.

## 2. Materials and Methods

### 2.1. Sample Preparation

The precursors employed for preparing pure CCTO and Sr-Zr co-doped CCTO ceramics with the formula Ca_0.8_Sr_0.2_Cu_3_Ti_4−*x*_Zr*_x_*O_12_ (*x* = 0.1, 0.2, 0.3, 0.4) samples were CaCO_3_ (99%, FUCHEN, Tianjin, China), SrCO_3_ (99%, FUCHEN, Tianjin, China), CuO (99%, FUCHEN, Tianjin, China), TiO_2_ (98%, Sinopharm Chemical Reagent Co., Ltd., Shanghai, China), and ZrO_2_ (99%, aladddin, Shanghai, China) powders. The powders were weighed according to the stoichiometric ratio and mixed by ball milling using agate grinding balls in ethanol for 6 h at 300 rpm under atmospheric conditions. The mixture was dried in an oven at 100 °C for 8 h under atmospheric conditions and then placed in an alumina crucible and sintered at 1000 °C for 3 h to obtain the synthesized powder. The synthesized powder was subjected to ball milling for 2 h and dried again. Subsequently, 10 wt% polyvinyl butyral (PVB) was added as a binder to the synthesized powder, and the mixture was pressed into pellets with diameters of 12.5 mm and thicknesses of 2–3 mm by uniaxial pressing at 150 MPa for 1 min. The disks were calcined in three stages at 400 °C for 1 h, 700 °C for 0.5 h, and 400 °C for 1.5 h to remove the binder. Finally, the green disks were calcined at 1020 °C for 6 h in a muffle furnace. For the sake of the survey of the dielectric properties of the samples, the surfaces on both sides of the pellets were polished and painted with a layer of silver paste to create capacitor electrodes and then sintered at 600 °C for 0.5 h in the air [32]. The as-prepared Ca_0.8_Sr_0.2_Cu_3_Ti_4−*x*_Zr*_x_*O_12_ samples with *x* values of 0.1, 0.2, 0.3, and 0.4 are denoted herein as SrZr1, SrZr2, SrZr3, and SrZr4, respectively.

### 2.2. Characterization Methods

The phase composition of the samples at room temperature was determined by X-ray diffraction (XRD) using a D8 Advance diffractometer (Bruker, Karlsruhe, Germany) in Bragg–Brentano geometry with Cu-Kα1 radiation (λ = 0.15406 nm) in a scanning range of 2θ = 20°–80° with a step size of 0.02°, which is provided by a secondary graphite monochromator. The microstructures of the samples were characterized by scanning electron microscopy (SEM) using a Quanta 200 microscope (FEI, Eindhoven, The Netherlands). X-ray photoelectron spectroscopy (XPS) was conducted using an ESCALAB 250Xi spectrometer (Thermo Fisher, Waltham, America) to evaluate the chemical states of the cations. Impedance and dielectric performance testing were conducted at room temperature using a Concept 80 broadband dielectric spectrometer (Novocontrol, Montabaur, Germany) with a frequency range of 1 Hz to 10^6^ Hz.

## 3. Results and Discussion

### 3.1. Phase Composition

**Figure 1:**
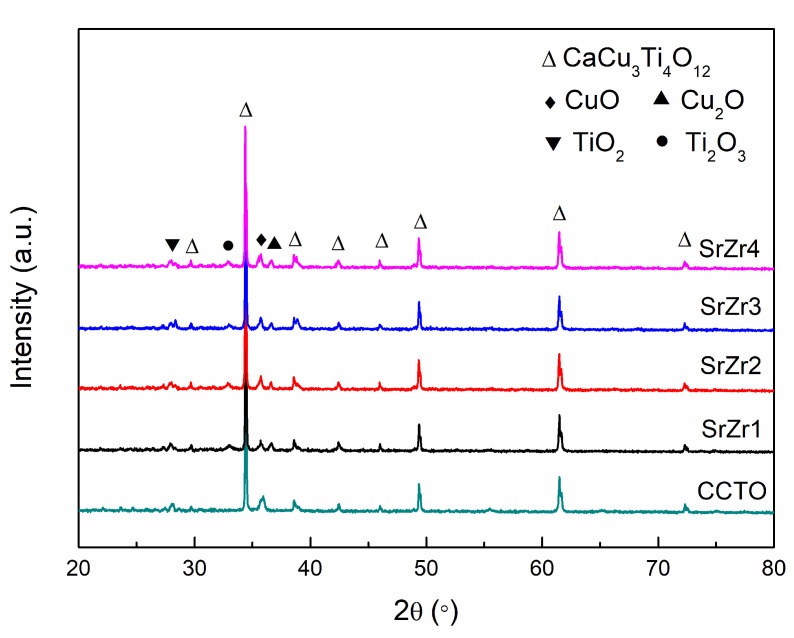
XRD patterns obtained for the as-prepared CCTO, SrZr1, SrZr2, SrZr3, and SrZr4 ceramics.

The phase compositions of the as-prepared CCTO, SrZr1, SrZr2, SrZr3, and SrZr4 samples can be determined according to the XRD patterns presented in Figure 1. We can see that the primary phase observed for all the samples is CaCu_3_Ti_4_O_12_, which is a body-centered cubic perovskite structure in the Im-3 space group (JCPDS 75-2188). Trace phases of CuO (JCPDS 80-1916), Cu_2_O (JCPDS 77-0199), TiO_2_ (JCPDS 82-0514), and Ti_2_O_3_ (JCPDS 85-0868) are also observed for the sintered samples, while no phases involving Sr and Zr are discernible in any of the XRD patterns. The lattice parameters determined from analyses of the XRD patterns obtained for the samples are listed in Table 1. We note that the lattice parameters present a slight increasing trend with the substitution of Sr and the increasing x of the Zr content from 0.1 to 0.4. This can be attributed to the fact that the ionic radii of Sr^2+^ (1.16 Å) and Zr^4+^ (0.72 Å) are, respectively, larger than those of Ca^2+^ (1.0 Å) and Ti^4+^ (0.605 Å) [33]. Accordingly, this increases the Ca-Cu and Ti-O bond lengths, which increases the lattice parameters of the materials [34].

### 3.2. Microstructure

**Figure 2:**
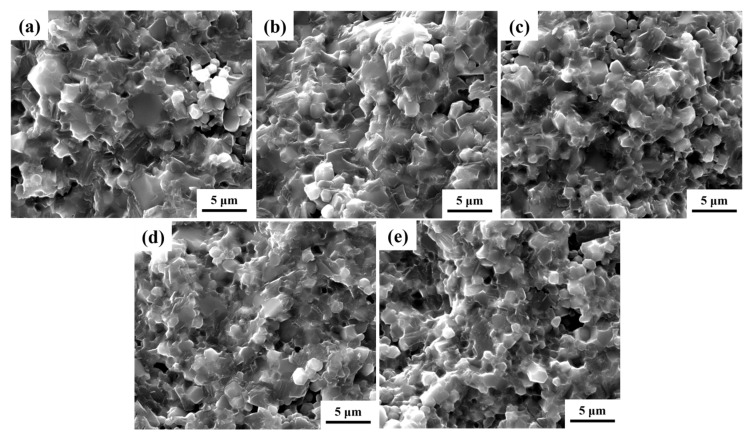
SEM images of the fractured surface of the as-prepared ceramics: (**a**) CCTO; (**b**) SrZr1; (**c**) SrZr2; (**d**) SrZr3; (**e**) SrZr4.

The microstructure of the samples can be determined from the SEM images obtained for fractured surface of the as-prepared CCTO, SrZr1, SrZr2, SrZr3, and SrZr4 samples presented in Figure 2a–d, and e, respectively. We note that all samples exhibit irregular grain shapes, and that the microstructure of the Sr–Zr co-doped CCTO ceramics differ from that of the pure CCTO ceramic. Analyses of the SEM images yields average grain size values for the CCTO, SrZr1, SrZr2, SrZr3, and SrZr4 ceramics of about 2.79 μm, 2.59 μm, 2.47 μm, 2.33 μm, and 2.39 μm, respectively. Accordingly, the grain size presents a decreasing trend with the substitution of Sr and the increasing Zr content of x from 0.1 to 0.4. The liquid phase exists in the high temperature sintering process of ceramics, which will promote the mobility of ions, and the diffusion of ions at the grain boundaries enhances the growth of grains. The decrease in grain size elucidates that Zr ions can inhibit the formation of the liquid phase, thus restricting the movement of grain boundaries and reducing the grain sizes [35]. Similar results have been reported previously [36].

### 3.3. Oxidation States of Cations

**Figure 3:**
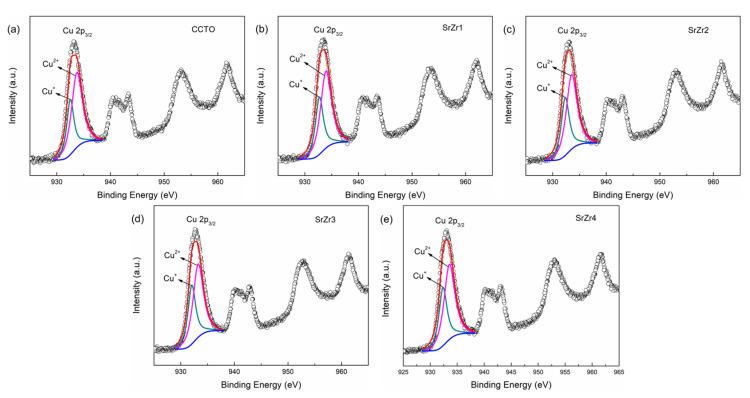
XPS Cu 2p spectra obtained for the as-prepared ceramics: (**a**) CCTO; (**b**) SrZr1; (**c**) SrZr2; (**d**) SrZr3; (**e**) SrZr4.

**Figure 4:**
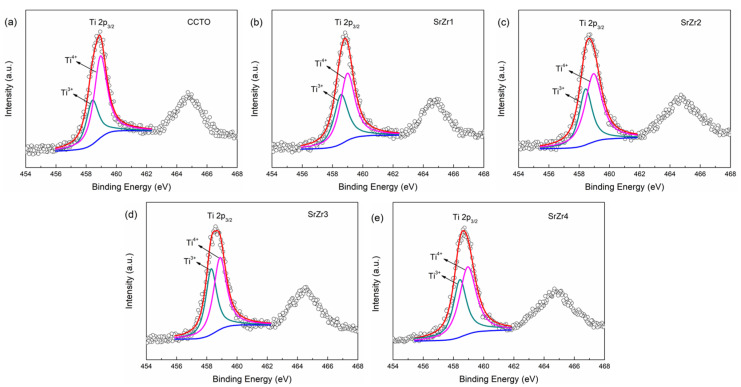
XPS Ti 2p spectra obtained for the as-prepared ceramics: (**a**) CCTO; (**b**) SrZr1; (**c**) SrZr2; (**d**) SrZr3; (**e**) SrZr4.

The oxidation states of the Cu and Ti cations can be analyzed according to the XPS Cu 2p and Ti 2p spectra presented in Figure 3 and Figure 4, respectively. In these analyses, the spectra associated with CCTO, SrZr1, SrZr2, SrZr3, and SrZr4 ceramics are presented in subfigures a, b, c, d, and e, respectively. The spectra were subjected to Shirley background subtraction, and the peaks were analyzed using Gaussian–Lorentzian curves for fitting [37]. Specifically, the asymmetric Cu 2p_3/2_ and Ti 2p_3/2_ peaks obtained for all samples were divided into two separate peaks each with binding energies associated with Cu^+^ and Cu^2+^ oxidation states and Ti^3+^ and Ti^4+^ oxidation states, respectively. This result is correspond to the detection results of XRD in doped samples. We note from the results in Figure 3a–e that the peaks associated with Cu^+^ have low binding energies ranging from 932.05 to 932.70 eV, while the peaks associated with Cu^2+^ have high-binding energies ranging from 933.23 to 933.93 eV. We notice from the results in Figure 4a–d, that the peak positions associated with Ti^3+^ have low-binding energies ranging from 458.19 to 458.77 eV, while the peaks associated with Ti^4+^ have high-binding energies ranging from 458.68 to 459.21 eV. The proportions of the Cu^+^/Cu^2+^ and Ti^3+^/Ti^4+^ oxidation states are listed in Table 2. The results present trends for both Cu and Ti, where the ratios of Cu and Ti atoms in Cu^+^ and Ti^3+^ oxidation states increase with the substitution of Sr and the increasing Zr content of *x* from 0.1 to 0.4. These results demonstrate that doping with Sr and Zr increases the degree of Cu^+^/Cu^2+^ and Ti^3+^/Ti^4+^ valence state mixing in CCTO ceramics.

The XPS results can be explained as follows. First, we note that the high-temperature sintering of ABO_3_ perovskite oxides generate oxygen vacancies according to the following reaction [38]:

(1) $$O_{o}\to \frac{1}{2}O_{2}+V_{o}^{••}+2e^{-}$$

where O_o_ represents an oxygen ion in the lattice, $V_{o}^{••}$ is an oxygen vacancy, and *e*^−^ is an electron. When oxygen vacancies are formed in the lattice, the charge carriers’ concentration will be increased, while Cu^2+^ and Ti^4+^ will capture the released electrons to produce Cu^+^ and Ti^3+^ for charge compensation [39,40]:

(2) $${\mathrm{Cu}}^{2+}+e^{-}\to {\mathrm{Cu}}^{+}$$

(3) $${\mathrm{Ti}}^{4+}+e^{-}\to {\mathrm{Ti}}^{3+}$$

The electrons can be transferred among Cu^2+^ and Cu^+^, and Ti^4+^ and Ti^3+^, which represents electron conduction. Accordingly, the crystalline grains of the samples are semiconducting, and CCTO ceramics are an n-type semiconductor [41,42]. The XPS results in Figure 3 and Figure 4 clearly display this mixed Cu^+^/Cu^2+^ and Ti^3+^/Ti^4^ valence structure. We further note that the lower electronegativity of Sr (0.95) and Zr (1.33) than Ca (1.0) and Ti (1.54), respectively, increases the loss of O_o_ in the Ca_0.8_Sr_0.2_Cu_3_Ti_4−x_Zr_x_O_12_ ceramics above that of the un-doped CCTO ceramic when sintering. Thus, the concentration of oxygen vacancies in the Ca_0.8_Sr_0.2_Cu_3_Ti_4−x_Zr_x_O_12_ ceramics is greater than that of the pure CCTO ceramic, which leads to a greater concentration of released electrons, and greater Cu^+^ and Ti^3+^ concentrations [43]. This is clearly demonstrated by the results presented in Table 2.

### 3.4. Dielectric Properties

**Figure 5:**
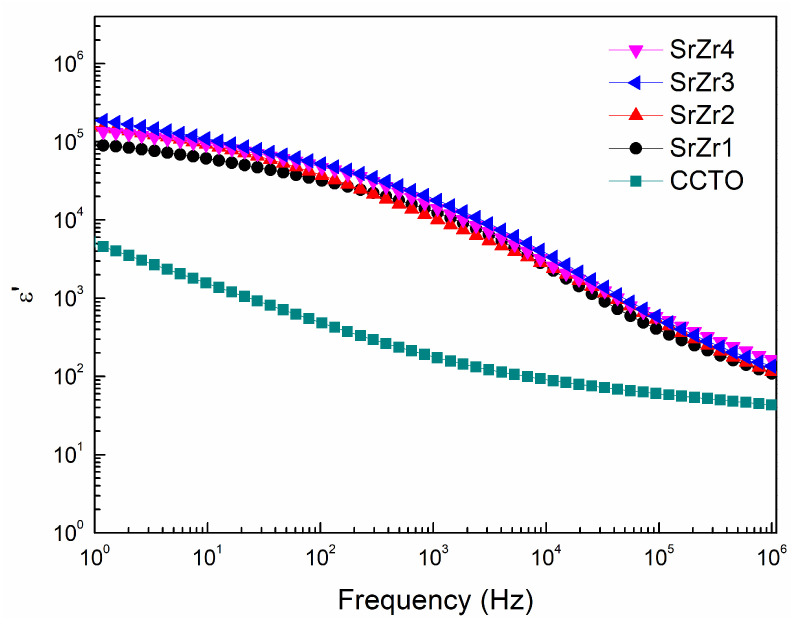
Frequency dependence of the dielectric constant (*ε*′) obtained for the CCTO, SrZr1, SrZr2, SrZr3, and SrZr4 ceramics.

The frequency dependence of the dielectric constant (*ε*′) obtained for the CCTO, SrZr1, SrZr2, SrZr3, and SrZr4 ceramics is presented in Figure 5, respectively. The results demonstrate that the values of *ε*′ obtained for all samples decreased with increasing frequency, and that the decrease was sharp for the co-doped ceramics in the frequency range of 10^4^ Hz to 10^6^ Hz. We further note that the Sr–Zr co-doped samples achieved greater dielectric constant values than the pure CCTO sample over the full range of frequency. Among the four co-doped samples, we find that the SrZr3 ceramic provided the maximum dielectric constant with a value as great as 1.87 × 10^5^ at 1 Hz, which is nearly 100 times greater than that provided by the pure CCTO ceramic (i.e., 4.57 × 10^3^ at 1 Hz). Meanwhile, the value of *ε*′ observed for the co-doped CCTO ceramics is greater than 10^4^ at a frequency less than 10^3^ Hz, and the co-doped ceramics therefore maintain higher *ε*′ values than the pure CCTO ceramic over a wide frequency range.

The value of *ε*′ obtained at a given frequency is positively correlated with the polarizability of the material at that frequency. Therefore, these results can also be explained in part by the mixed Cu^+^/Cu^2+^ and Ti^3+^/Ti^4+^ valence structure, which forms dipoles that promote dipole polarization and thereby increases the dielectric constant of the samples [44]. In addition, the polarizability of Sr^2+^ (0.861) and Zr^4+^ (0.357) is greater than that of Ca^2+^ (0.469) and Ti^4+^ (0.184), respectively [45]. Thus, doping with Sr and Zr ions substantially increases the values of *ε*′ obtained for the co-doped CCTO ceramics.

**Figure 6:**
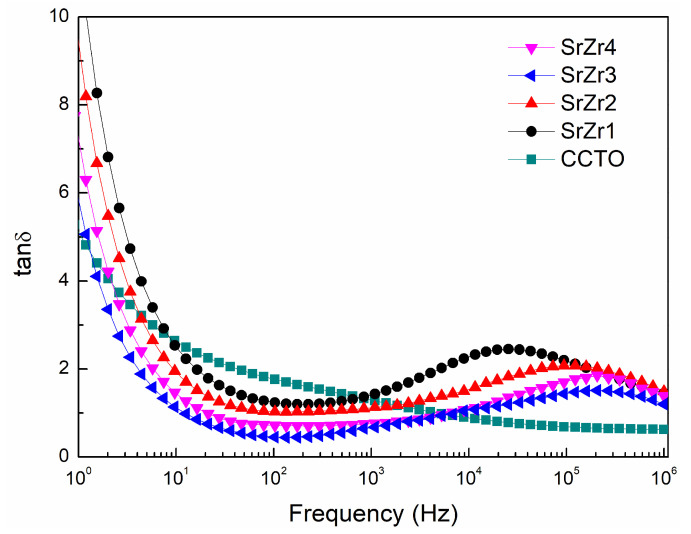
Frequency dependence of the dielectric loss (tanδ) obtained for the CCTO, SrZr1, SrZr2, SrZr3, and SrZr4 ceramics.

The dielectric loss (tanδ) obtained for the CCTO, SrZr1, SrZr2, SrZr3, and SrZr4 ceramics over the frequency range of 1 Hz to 10^6^ Hz are presented in Figure 6. We can clearly see that the dielectric loss of the pure CCTO ceramic decreases gradually with the increasing frequency over the entire frequency range. In contrast, the dielectric loss of the Sr–Zr co-doped ceramics initially decrease rapidly with the increasing frequency down to minimum values at frequency on the order of 10^2^ Hz. Here, we note that the minimum dielectric loss obtained first decreases then increases with increasing Zr concentration, and the SrZr3 ceramic obtains the lowest loss (0.43, 10^2^ Hz) among all samples considered, while the dielectric constant is still as high as 4.80 × 10^4^ at 10^2^ Hz. Subsequently, the dielectric loss of the co-doped ceramics increases to local maximum values much greater than that of the pure CCTO ceramic at frequency on the order of 10^5^ Hz. The peaks in the tanδ plots observed for the co-doped CCTO ceramic samples at high frequency can be attributed to the typical Debye relaxation phenomenon [46], which explains the decrease in *ε*′ observed in Figure 5 for the co-doped ceramics in the frequency range of 10^4^ Hz to 10^6^ Hz. This relaxation phenomenon arises due to the fact that the dipoles formed by the mixed Cu^+^/Cu^2+^ and Ti^3+^/Ti^4+^ valence structure in the materials become increasingly unable to synchronize with the changing of the applied electric field with increasing frequency.

### 3.5. Impedance Spectroscopy

**Figure 7:**
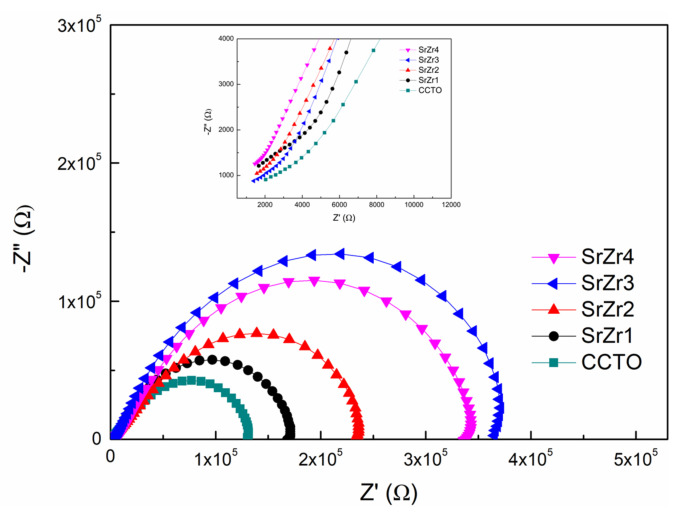
Impedance spectra of CCTO, SrZr1, SrZr2, SrZr3, and SrZr4 ceramics, where the inset presents an enlarged view near the origin.

**Figure 8:**
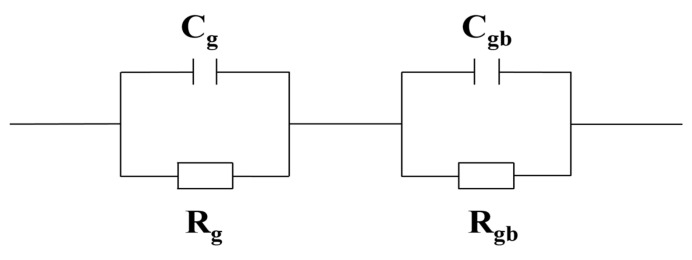
Equivalent circuit used for establishing the parameters of the IBLC model.

The impedance spectra (−Z′′ versus Z′) obtained for the SrZr1, SrZr2, SrZr3, and SrZr4 ceramics are presented in Figure 7, where the inset presents an enlarged view near the origin. These results can be analyzed according to the IBLC model based on the equivalent circuit presented in Figure 8, which consists of two components R_gb_C_gb_ and R_g_C_g_ composed of resistance R and capacitance C in parallel that are connected in series [47]. Here, R_gb_C_gb_ represents the insulating grain boundaries and R_g_C_g_ represents the semiconducting grains. The value of R_gb_ can be estimated from the intercepts of the two arcs on the Z′ axis, and the value of R_g_ can be obtained from the nonzero intercept on the Z′ axis [48]. The values of R_gb_ and R_g_ are listed in Table 3. The results demonstrate that the values of R_g_ are uniformly two orders of magnitude less than the values of R_gb_ for all Sr–Zr co-doped ceramics. Accordingly, the grains are semiconducting, and the grain boundaries are insulating, which is consistent with the electrical heterogeneity assumed by the IBLC model. Meanwhile, the value of R_g_ decreases and the value of R_gb_ increases with increasing Zr concentration, and the lowest R_g_ (1.3 × 10^3^ Ω) and the highest R_gb_ (3.7 × 10^5^ Ω) are obtained for the SrZr3 ceramic. Accordingly, we can conclude that Zr substitution at Ti sites increases the conductivity of the crystalline grains of the Sr-doped CCTO ceramics, while simultaneously increasing the electrical resistance of the grain boundaries. The decrease in R_g_ with increasing Zr content reflects the increasing concentration of oxygen vacancies, which leads to a greater concentration of released electrons and greater electrical conductivity. The increase in R_gb_ may be attributable to the doping of Zr ions, which leads to a decreasing average grain size that increases the density of grain boundaries and ultimately increases the grain boundary resistance [49].

The dielectric loss results in Figure 6 can be now analyzed further by noting that the value of tanδ is inversely proportional to R_gb_ at a low frequency (10 Hz–10^3^ Hz), and can be estimated according to the following equation:

(4) $$\tan\delta \approx \frac{1}{\omega R_{gb}C_{p}}$$

where *ω* is the angular frequency of the excitation signal and *C_p_* is the electric capacity of the ceramic, which is positively correlated with *ε*′ [50]. Thus, we can conclude from Equation (4) that low tanδ values are associated with large values of R_gb_*C_p_*. Therefore, assuming that *C_p_* remains constant for the four Sr–Zr co-doped samples, the dielectric loss of the ceramics should first decrease with increasing Zr concentration, and then increase after the Zr content reaches 0.3, as observed in Figure 6.

## 4. Conclusions

The present work investigated the effects of Sr and Zr co-doping on the dielectric properties of CCTO ceramics by preparing pure CCTO and a series of Sr-Zr co-doped CCTO ceramics with the formula Ca_0.8_Sr_0.2_Cu_3_Ti_4−*x*_Zr*_x_*O_12_ (*x* = 0.1, 0.2, 0.3, 0.4) via a solid-state reaction technique. The effects of the Zr concentration on the phase composition, microstructure, cationic valence states, impedance, and dielectric properties of the as-prepared samples were examined in detail. The results demonstrated that all samples were composed of a cubic perovskite structure, and the lattice parameters obtained for the co-doped materials were greater than those of pure CCTO. The dielectric properties results show that the Sr and Zr co-doping process increases dielectric constant and reduces dielectric loss simultaneously, and the maximum dielectric constant (1.87 × 10^5^, 1 Hz) and minimum dielectric loss (0.43, 10^2^ Hz) were obtained when *x* = 0.3. The increased dielectric constant of the co-doped ceramics could be attributed the dipole polarization promoted by the mixed Cu^+^/Cu^2+^ and Ti^3+^/Ti^4+^ valence states observed to coexist in the co-doped material lattices. Application of the IBLC model demonstrated that Zr substitution at Ti sites increases the conductivity of the crystalline grains of the Sr–Zr co-doped CCTO ceramics, while simultaneously increasing the electrical resistance of the grain boundaries. The maximum grain boundary resistance (3.7 × 10^5^ Ω) was obtained for *x* = 0.3, which was the primary factor contributing toward the minimum dielectric loss obtained for this ceramic at a frequency less than 1 kHz. This could be explained in accordance with the effect of Zr doping on the average grain sizes of the samples, which decreased with increasing Zr content.

## Figures and Tables

**Table 1:** Structure parameters of the pure CaCu_3_Ti_4_O_12_ (CCTO) and Sr–Zr co-doped Ca_0.8_Sr_0.2_Cu_3_Ti_4−*x*_Zr*_x_*O_12_ (*x* = 0.1, 0.2, 0.3, 0.4) ceramics.

Samples	CCTO	SrZr1	SrZr2	SrZr3	SrZr4
Lattice parameter (Å)	7.376	7.378	7.379	7.381	7.382

**Table 2:** Fitting results of XPS Cu 2p_3/2_ spectra and Ti 2p_3/2_ spectra for CCTO, SrZr1, SrZr2, SrZr3, and SrZr4 ceramics.

Samples	CCTO	SrZr1	SrZr2	SrZr3	SrZr4
Cu^+^/Cu^2+^ (%)	31.35	34.42	35.35	37.01	36.67
Ti^3+^/Ti^4+^ (%)	34.05	38.39	41.11	43.75	42.36

**Table 3:** Values of R_g_ and R_gb_ respectively associated with grains and grain boundaries obtained for the CCTO, SrZr1, SrZr2, SrZr3, and SrZr4 ceramics.

Samples	CCTO	SrZr1	SrZr2	SrZr3	SrZr4
R_g_ (Ω)	2.0 × 10^3^	1.6 × 10^3^	1.5 × 10^3^	1.3 × 10^3^	1.4 × 10^3^
R_gb_ (Ω)	1.3 × 10^5^	1.7 × 10^5^	2.4 × 10^5^	3.7 × 10^5^	3.3 × 10^5^
